# Plasma “bullet” with hollow structure: formation and evolution

**DOI:** 10.1038/s41598-018-25962-z

**Published:** 2018-05-15

**Authors:** Zhengshi Chang, Ni Zhao, Guoqiang Li, Guanjun Zhang

**Affiliations:** State Key Laboratory of Electrical Insulation and Power Equipment, The School of Electrical Engineering, Xi’an Jiaotong University, Xi’an, 710049 P. R. China

## Abstract

Since the plasma “bullet” and ring shape were discovered by Teschke and coworkers in 2005, the hollow structure of the plasma “bullet” has been a hot topic as an important phenomenon. Clearing the mechanism back on the phenomenon is very important to research and application of atmospheric pressure plasma jet (APPJ). Although a lot of discussions on the generation and evolution of the hollow structure have been conducted in past years, there is a substantial divergence between the experimental researcher and the numerical simulation researcher. The former considers that the Penning effect has a main contribution, because the presence of impurities enables the Penning process to occur at the gas flow/air interface. On the contrary, numerical simulation claims that the Penning effect is not so decisive to the formation of hollow structure. Based on our previous work, this paper aims to clear the debatable topic by setting the special experiments. After comparing and analyzing the phenomena and mechanism, a better comprehension is reached on the contribution of the Penning effect to the hollow structure. We also give a promising conclusion for forming the hollow structure of plasma jet in the end of paper.

## Introduction

2005, Teschke and coworkers^1^ found the plasma “bullet” and the ring shape in He APPJ driven by AC with kHz power source. As an intriguing phenomenon, it has been extensively investigated in recent years since then^2–16^. Lu *et al*.^2^ also observed the plasma “bullet” in case of a nanoseconds pulsed jet, and gave an explanation to the propagation of the plasma “bullet” by building a model based on the photo-ionization. Subsequent many works, which can approximately be classified into two categories - experiment^2–10^ and numerical simulation^6,11–16^ - focus on the characteristics and the mechanism of the special phenomenon. Actually, these works contribute a lot (especially for freshmen) to understanding of the ignition, propagation and basic characteristics as well as partially mechanism. However, an opposite opinion on the physical mechanism of the formation continuously exists. Until our previous work was opened^9^, experimental researchers usually believed that the hollow structure of plasma “bullet” is closely related to the Penning effect on the interface of He/Air. For instance, Karakas *et al*.^4^ and Mericam-bourdet *et al*.^3^ got a plasma “bullet” exhibiting a hollow donut-shape and believed that solitary surface ionization waves should be responsible for the creation of the “bullets”. Urabe *et al*.^5^ deduced the radial saddle distribution for absorption peak of He* captured by employing laser absorption spectra and concluded that the streamer discharge happens on the interface between He channel and air ambient. Sakiyama *et al*.^6^ thought that air diffusion against helium convection creates the unique ring-shape emission profile in plasma “bullets” by employing experimental observation and finite analysis. Leiweke *et al*.^7^ observed ring shapes of several lines in both APPJ with pure Helium and mixture of Helium +5% Argon, and found that the cross section images 4 mm outside nozzle of the two APPJs transit from ring-shape into a solid when 5% Argon added into the pure Helium channel. Wu *et al*.^8^ also arrived at the discussion that the Penning effect has a primary role when a little of nitrogen gas (15sccm) is mixed into the pure helium (1slm) channel. Recently, Xian *et al*.^10^ designed several experiments to discuss the influence factors on the ring shape of plasma “bullet” in pin-to-pin electrode structure by some digital photos.

On the contrary, most of the numerical simulation workers convinced that Penning effect should not be a primary contribution on the hollow shape of plasma “bullet”, because typical parameters always present even if they threw away the Penning reaction from their model. For instance, series of works of Naidis^11,12^ by employing a 2D streamer model indicated that the ring shape of plasma “bullet” mainly depends on the initial electron density distribution, while Penning process just has a quantitative effect on the parameters of the streamer. It was considered in his recent work^16^ that the ring shape should be related to the radial non-uniformity of direct impact ionization rate between gas species and electron. By applying one positive square pulse voltage without repetitive frequency, Breden *et al*.^13,14^ got the conclusion that the ring shape forms mainly due to electric field distribution and the electron-impact with He and N_2_ at the interface of pure helium and air, while the penning effect just contributes to the increase of the streamer propagation speed with the photoionization. Boeuf *et al*.^15^ gave a detailed interpretation on the plasma “bullet” and its structure based on the comparison between guided streamer and cathode directed streamer. The results shown that the “helium tube” can form a sharp discontinuous interface between helium and ambient air which should be a main factor to generate the ring-shape of plasma “bullet”.

Based on above review, obviously, a highlighted disagreement lies between experimental and numerical simulation researches. To resolve this argument and clarify the formed mechanism of the hollow shape of plasma “bullet” have became urgent things. Some further works are necessary for understanding well about the hollow shape and other further unknowns of atmospheric pressure plasma “bullets” and jets.

In our previous work^9^, we tried to interpret the abovementioned disagreement by providing some convincing experimental evidences. Based on it, in this paper, we would like to give a further demonstration and analysis to understand the formation and evolution of plasma “bullet” with hollow structure.

## Results

**Figure 1:**
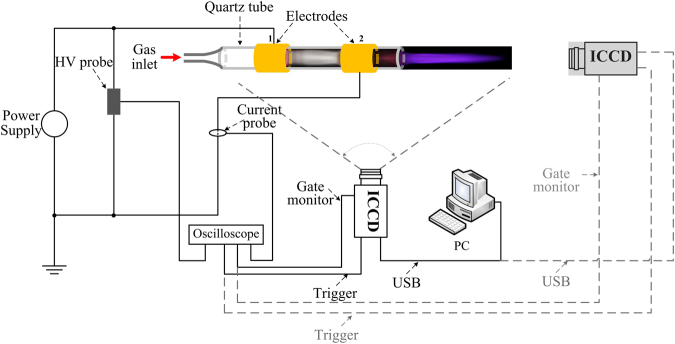
Experimental schematic.

In order to achieve the goal, we set several experiments in this paper. The experimental sketch is shown in Fig. 1.

**Figure 2:**
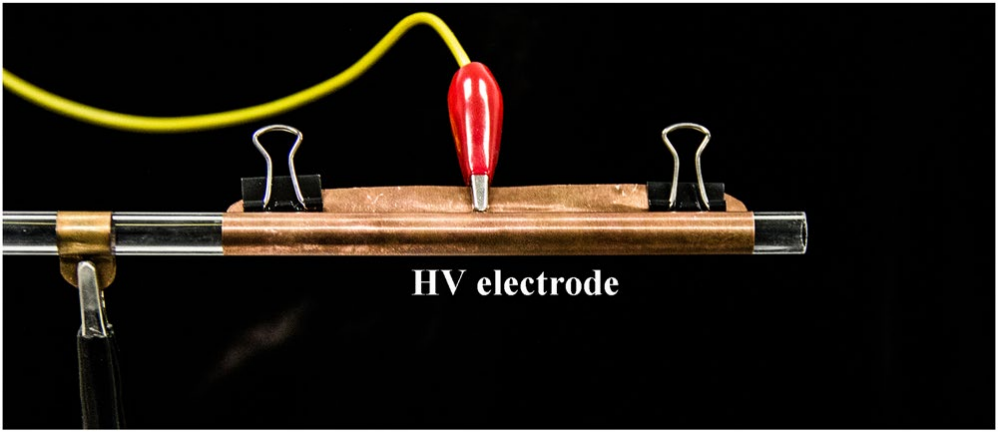
Experimental electrode configuration.

**Figure 3:**
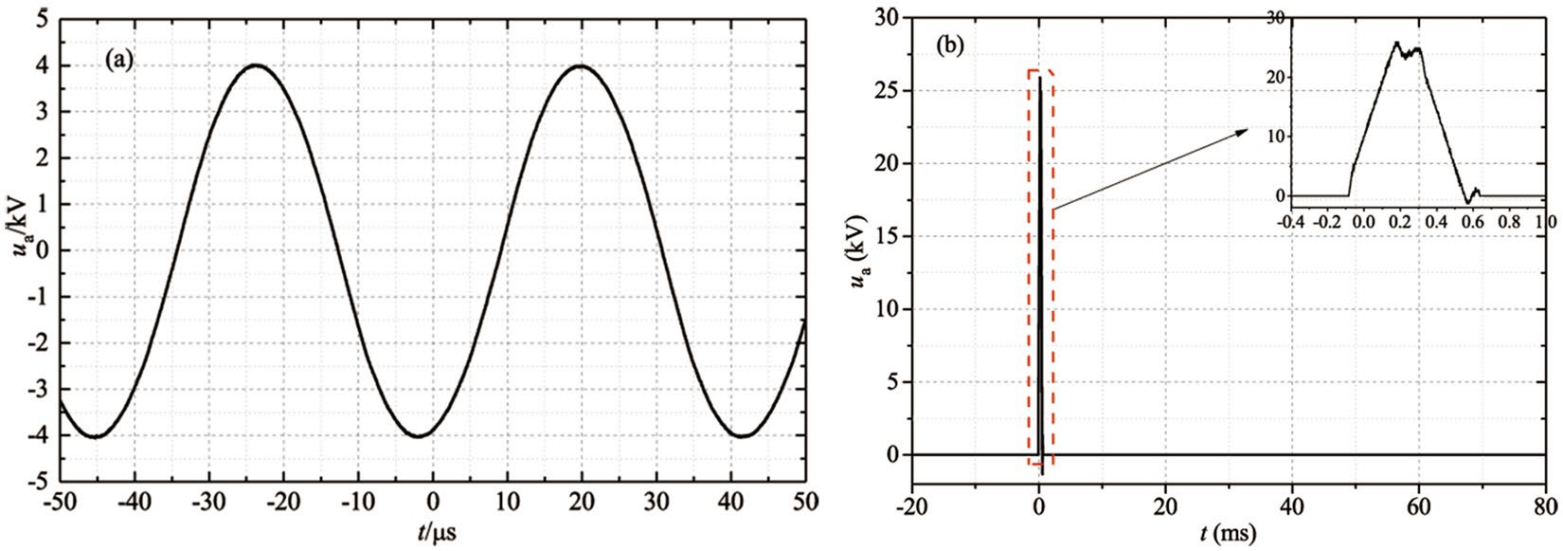
Applied voltage waveform: (**a**) AC with the frequency of 23 kHz. (**b**) Positive pulse with the frequency of 0.5 Hz.

The dynamic images of the plasma “bullet” were captured by two ICCDs with the same model (Andor iStar 334T) which were synchronously triggered. The current and voltage signals were taken by employing a current probe Pearson 2877 and high voltage probe P6015A and were recorded with a digital oscilloscope. The electrode structure is shown in Fig. 2 which is the same as the electrode system used in our previous work^9^. The wider HV electrode with width of 10 cm is mainly used to eliminate the influence of discharge in DBD zone. First of all, the effects of size and the cross section shape of the dielectric tube on the structure of the plasma “bullet” are discussed. Then, the “bullet” in He APPJ driven by an AC power with 23 kHz of frequency (see Fig. 3(a)) was investigated because Penning effect is inevitable there. Further, a positive pulse voltage with 0.5 Hz of frequency (see Fig. 3(b)) was employed to generate a single discharge per single shot, where Penning effect can be effectively avoided because the active products in discharge will disappear during 2s of a cycle. At the same time, different ratio of impurity N_2_ was added into the pure helium gas (analytical purity, 5N) channel in two experiments for knowing evolution of the hollow structure. In the end of the paper, supplementary material provides another experiment as one more example evidence which was conducted in Ar/NH_3_ APPJ (Supplementary Information).

### Hollow structure of plasma “bullet” with different tube size and cross section shape

For the convenience of analysis, we proposed two terms “micro-hybrid interface zone” and “Penning ionization zone”. The former should stay between the carrier gas channel and ambient air due to the diffusion, entrainment and viscosity. The latter is defined as a zone with suitable mole fraction of He for Penning ionization. According to our previous work^17^, we compared the results of the experiment and the fluid simulation with and without APPJ. Mole fraction of helium in “Penning ionization zone” should follow a range from 98.7% to 99.8%. Therefore, micro-hybrid interface zone, which relies on the tube size and Reynold number (flow state), can’t be obviously changed in this paper. However, Penning ionization zone will be shrunk towards axis of gas channel because the mole fraction of helium is changed with different adding ratio of nitrogen into helium.

#### Different tube’s diameter

**Figure 4:**
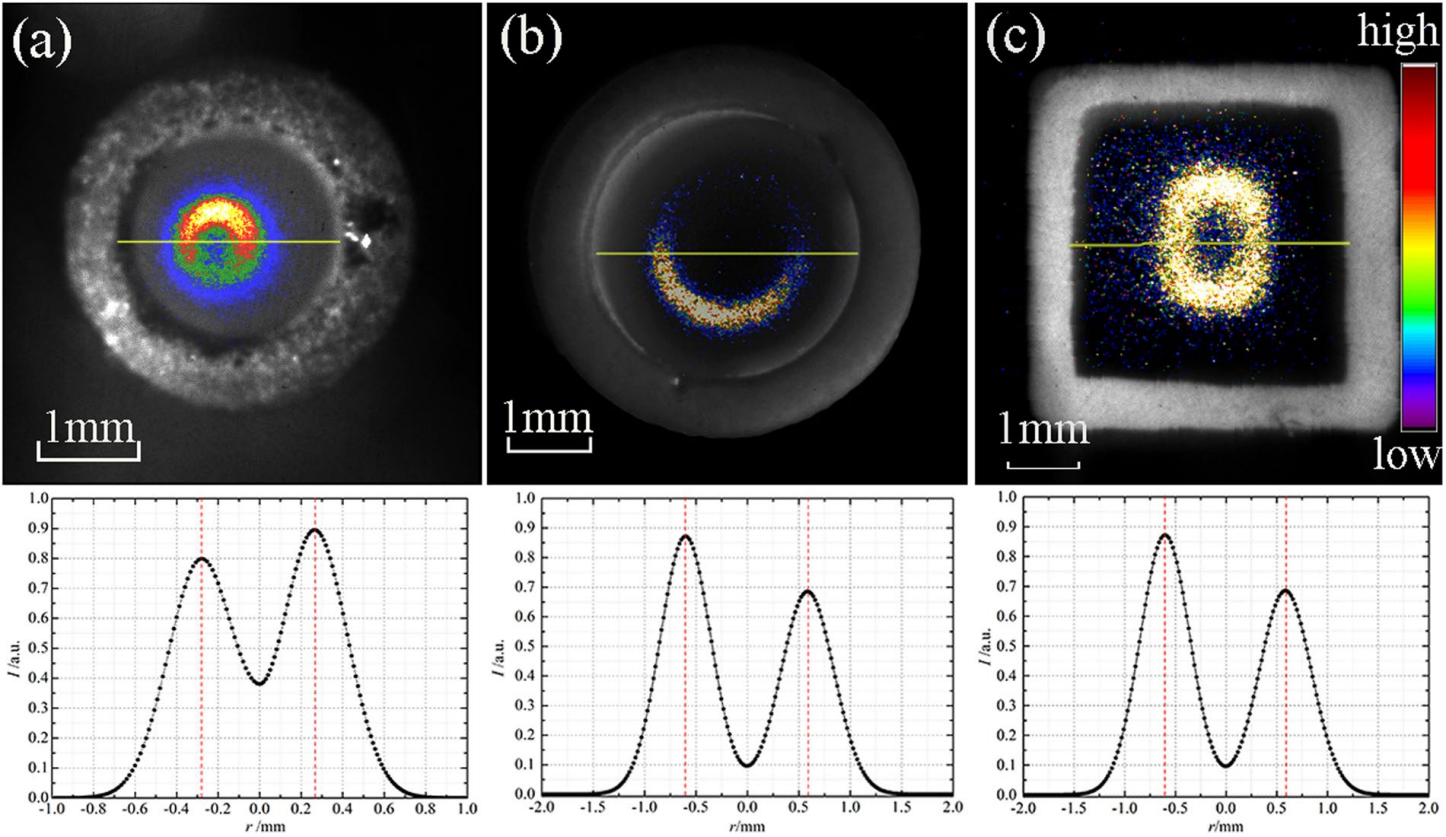
ICCD Images displayed in pseudo-color of different tube with normalized 1D lightness distribution: (**a**) glass tube of d = 2 mm, (**b**) glass tube of d = 4 mm, (**c**) ABS tube of L = 4 mm, exposure time: 5 ns.

Two size (diameter *d* = 2 mm and *d* = 4 mm) quartz tube (*ε* = 3.5–3.7) were employed. The ICCD images with 5 ns exposure time at 10 mm outside the tube nozzle are shown in Fig. 4(a,b). The front view images are the hollow structure in both case of 2 mm (top panel in Fig. 4(a)) and 4 mm (top panel in Fig. 4(b)) tube’s inner diameter. The curves plotted in bottom panel in each image are normalized 1D lightness distribution along the solid yellow line on radial, respectively. From the curves, the hump shape presents in two cases, and it can further be found that the maximum of light intensity appears at *r* = 0.28 mm for *d* = 2 mm tube and *r* = 1.20 mm for *d* = 4 mm tube. Therefore, the hollow ring-shape structure of plasma “bullet” should have a minor dependence on the size of dielectric tube when the size is no less than a certain value (such as 0.5 mm of tube inner diameter) in this paper (more systematic experiments around the relation of tube diameter and the hollow structure not shown here). And another recalled things is that the conclusion gotten by Naidis in 2010 might be reassessed in which a solid shape plasma “bullet” existed in case of 0.25 cm radius of tube^11^.

#### Different tube’s cross section shape

Also, to compare with the case of circle cross section shape, a ABS (Acrylonitrile–Butadiene–Styrene copolymer) tube (*ε* = 2.2–2.5) with square cross section of 4 mm length was used as a dielectric material to generate a helium plasma jet driven by a power the same as aforementioned. If an ICCD was focused at the position of 10 mm away from the tube nozzle, we can get an instantaneous image of plasma “bullet” in a hollow square shape, as shown top panel in Fig. 4(c). Analogously, a normalized 1D lightness distribution along with the solid yellow line was drawn in bottom panel. A hump shape is, obviously, obtained and the lightness peak appears at *r* = 0.6 mm.

The hollow structure of plasma “bullet” always presents whatever ring-shape or square-shape, not the size (when the “micro-hydro interface zone” and “Penning ionization zone” can be identified) and materials of tube. It is consistent with the analysis from Breden^13^ where dielectric with higher breakdown strength (just as a solid dielectric or air gas) exists around the helium gas channel and forms a guided gas channel. They can influence the propagation of the plasma “bullet” just like a guided streamer but not a common streamer.

### Penning effect versus hollow structure

In this section, contribution and influence of the penning effect on the hollow structure of helium plasma “bullet” were obtained by controlling the power types and added ratio of N_2_ in experiment. In order to clearly observe the structure of the plasma “bullet”, a quartz tube with inner/outer diameters of 5.5 mm/8 mm was used here. To synchronously capture the plasma “bullet” images from side view and front view, two ICCDs in the same model and with the same parameters sets were placed in orthometric directions as shown in Fig. 1.

#### Plasma driven by AC with 23 kHz

**Figure 5:**
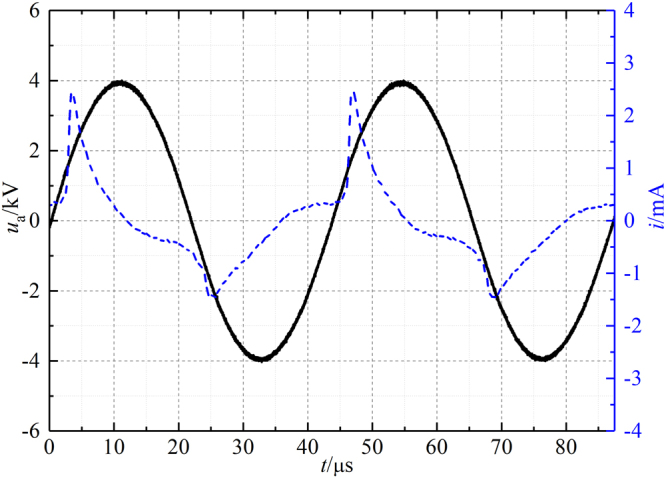
Current and applied AC voltage waveforms.

In case of AC power source with 23 kHz frequency, called “continuous discharge” here, the applied voltage (Fig. 3(a)) and the discharge current waveforms are given in Fig. 5.

**Figure 6:**
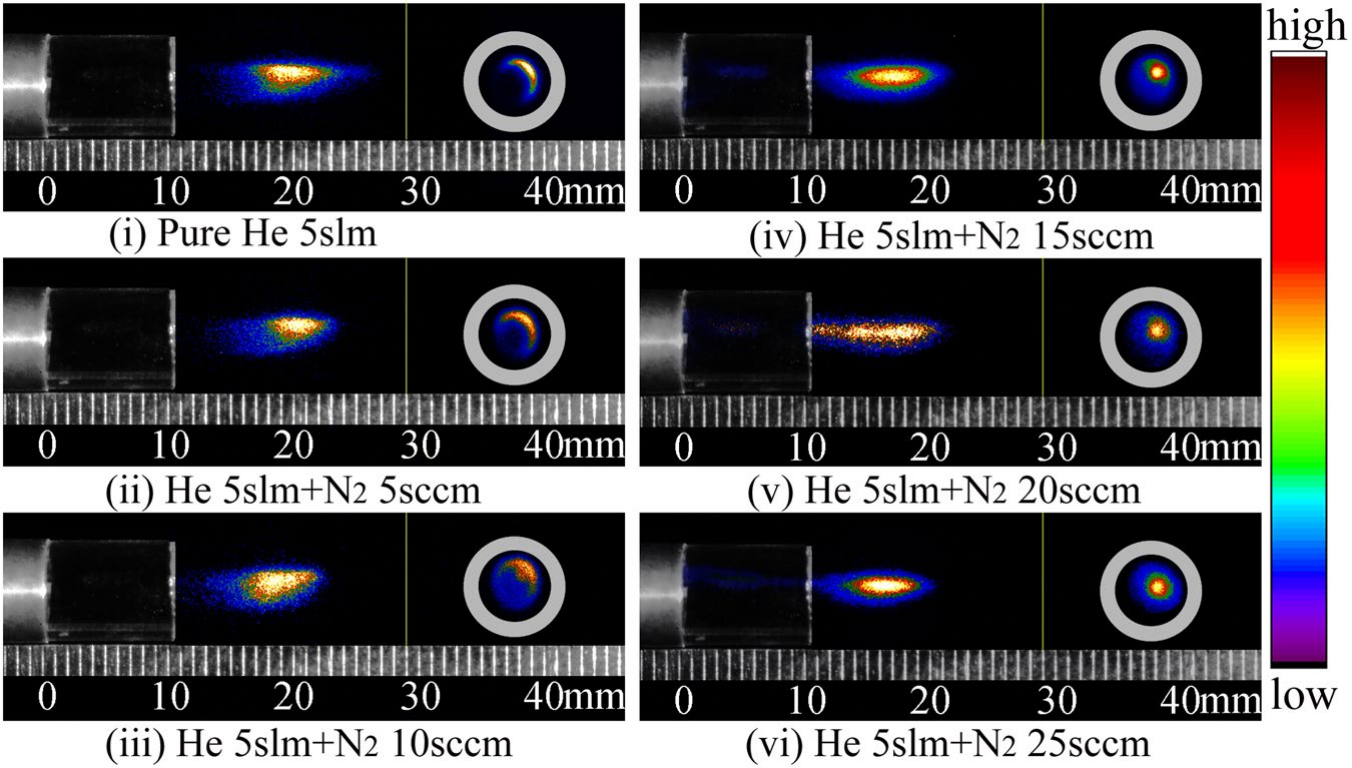
Side- and front-view in pseudo-color of plasma “bullet” driven by an AC power with 23 kHz of frequency, exposure time: 5 ns.

The images of plasma “bullet” with/without impurity N_2_ were captured by ICCDs and composited by image treatment software Photoshop^©^, shown in Fig. 6. In each image in pseudo-color, the side-view and the front-view photos of the “bullet” at the same moment and position are respectively shown on left and right sides of the yellow solid line. The ratio of added nitrogen gas covers from 0sccm to 25sccm with changing step of 5sccm. Figure 6 presents a hollow structure captured in case of pure helium and a little impurity N_2_ added (such as N_2_ flow rate of 5sccm). The front-view photo gradually transits from hollow into solid shape when the ratio of N_2_ exceeds a certain value, such as 10sccm.

#### Plasma driven by positive pulse with 0.5 Hz

As well known, Penning reaction happens between the metastable atomic He, produced in previous discharge, and some N_2_ whose ionization energy is slightly less than excited energy of the metastable atoms. It can provide more seed electrons for discharge. So it is difficult to avoid Penning effect in case of high frequency continuous discharge.

**Figure 7:**
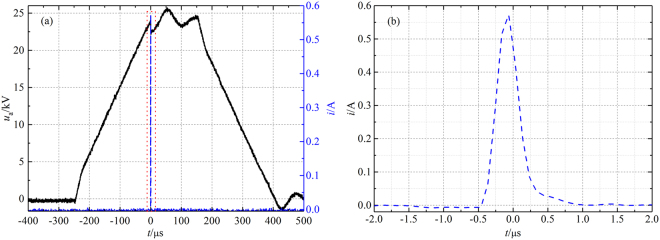
Current and applied pulsed voltage waveforms (**a**), and enlarged current waveform (**b**).

In order to eliminate this influence, a positive pulse with width of 400 *μ*s is written with an arbitrary waveform generator and is outputted after amplified by a high voltage amplifier, as shown in Fig. 3(b). Only single discharge (namely one current pulse per voltage pulse) was generated during this positive pulse, whose current waveform is shown in Fig. 7(a,b). Because of non-repeatability of single discharge, it is very difficult and time-consuming to get the ICCD image of plasma “bullet” with head just located at around 10 mm outside the nozzle. Therefore, the specific operates procedure described in *Section Methods* needs to be followed in oder to achieve this aim.

**Figure 8:**
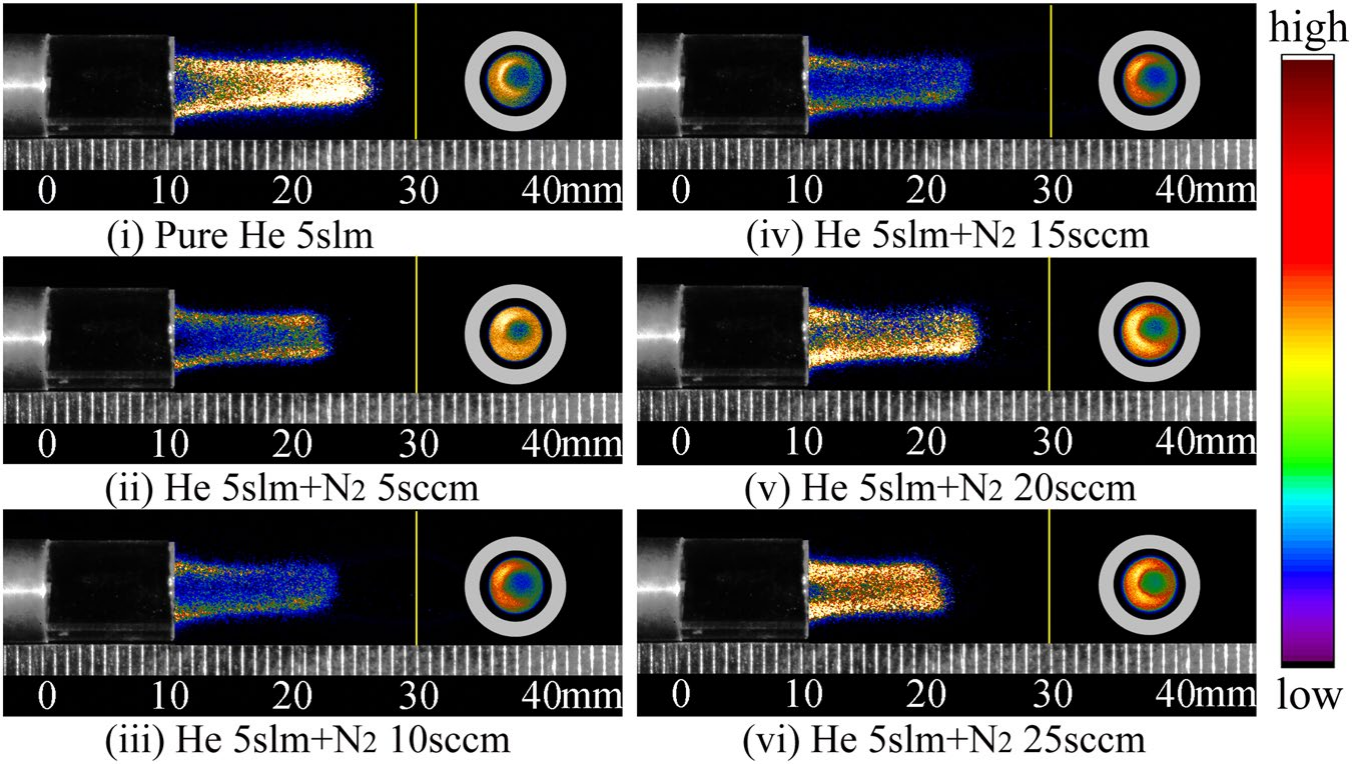
Side- and front-view in pseudo-color of plasma “bullet” driven by an pulse power with 0.5 Hz of frequency, exposure time: 2 ns.

The axial and radial ICCD images in pseudo-color for plasma “bullet” with 2 ns exposure time at 10 mm outside the nozzle, captured during single discharge and with different N_2_ addition, are shown in Fig. 8. Both of the images respectively locate on the left and right side of the yellow vertical bar in each figure. It can be seen that all the radial images of plasma “bullet” generated by single discharge present a relatively homogeneous hollow structure at 10 mm outside the nozzle, whatever for pure He or with N_2_ addition. Compared with high frequency continuous discharge described in last section, hollow structure in the single pulse discharge always exists, being not affected by N_2_ ratio, as shown in Fig. 8.

## Discussion

**Figure 9:**
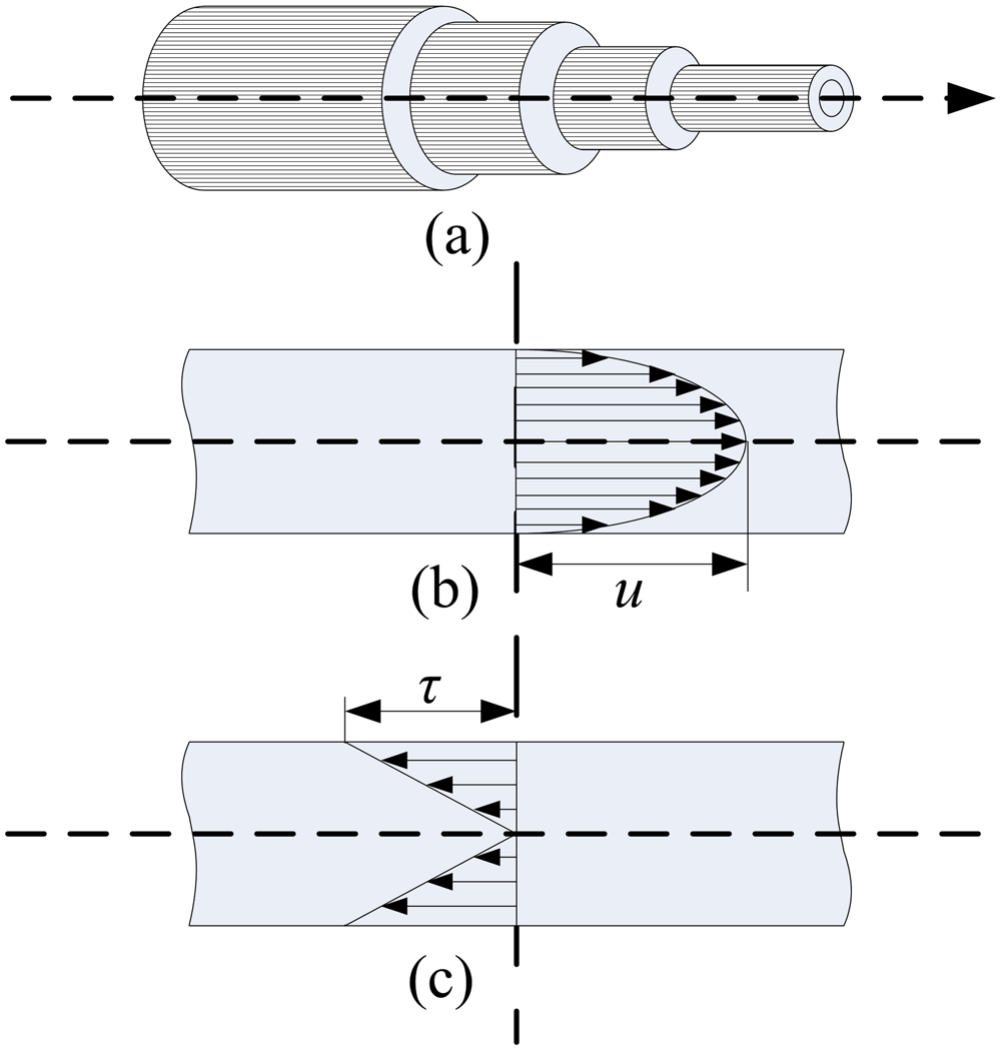
Schematic of laminar flow inside circular tube. (**a**) laminar model; (**b**) velocity distribution; (**c**) shear stress distribution.

On basis of the above experimental results, we give some discussions as follows. We will firstly discuss the gas flow status. Reynold number (*R*_e_), a typical parameter for expressing the status of fluid, can be calculated according to the definition of fluid in tube as follow. It is usually expressed as formula ${R}_{{\rm{e}}}=\frac{\rho {D}_{{\rm{H}}}u}{\mu }=\frac{Q{D}_{{\rm{H}}}}{\nu A}$, where *D*_H_ is the hydraulic diameter of the pipe (the inside diameter if the pipe is circular) (m), *Q* is the volumetric flow rate (m^3^s^−1^), *A* is the pipe’s cross-sectional area (m^2^), *u* is the mean velocity of the gas (ms^−1^), *μ* is the dynamic viscosity of the gas (kg (m ⋅ s)^−1^) (for helium gas, *μ* = 1.9561 × 10^−5^ kg (m ⋅ s)^−1^), *ν* is the kinematic viscosity ($\nu =\frac{\mu }{\rho }$ (m^2^s^−1^), *ρ* is the density of the fluid (kgm^−3^) (for helium gas, *ρ* = 0.16674 kgm^−3^). For helium gas flow inside glass tube, it is a laminar flow because the *R*_e_ is nearly equal to 164 which is far less than the critical value (generally, laminar flow presents when *R*_e_ < 2320 while the turbulent flow occurs when *R*_e_ > 2600, and the transition flow appears when *R*_e_ is between 2300 and 2600). The fluid at position a few centimeters downstream the nozzle can also be deemed as inside tube because the air ambient can act as a “tube”, which can be justified from the schlieren image in experiment^17,18^ and from the velocity and species distributions in fluid simulation^17^. Therefore, it means that the helium gas flow is completely laminar flow in our experiment. Based on the description in fluid mechanics, the laminar model of flow status, the velocity distribution and the shear stress distribution inside circular tube can typically be shown in Fig. 9(a–c), respectively. Under laminar flow, the gas flow just like a set of countless thin cylinders (Fig. 9(a)). The higher the velocity is, the smaller diameter of the cylinder is. On the edge of gas channel, the flow rate is close to zero because of shear stress (Fig. 9(b,c)). Therefore, in our experiment, (1) the analytic purity helium gas with laminar flow is injected into the ambient air, forming a helium channel surrounded by air. (2) A “micro-hybrid interface zone” is built at the interface between helium channel and air due to diffusion, entrainment and viscosity.

When we apply continuous voltage with high frequency, such as AC power with 23 kHz of frequency, (1) A lot of metastable He atoms with ∼10 *μ*s lifetime generated in previous discharge will live inside the whole gas channel and have Penning reaction with the N_2_ molecule following the main reactions such as, He_*m*_ + N_2_ → He + ${{\rm{N}}}_{2}^{+}$ + e, k = 7 × 10^−11^ cm^−3^s^−1^; He_*m*_ + O_2_ → + ${{\rm{O}}}_{2}^{+}$ + e, k = 2.5 × 10^−10^cm^−3^s^−1 ^^19^. It can produce abundant seed electrons for generating subsequent discharge. This process will make the discharge happen in this zone firstly. (2) The reactions, such as excitation and re-excitation, are frequent in that zone, leading to an optical ring-shape. (3) By increasing N_2_ in helium gas channel, the mole distribution of He atom and N_2_ molecule was meanwhile changed. When the ratio of N_2_ reaches a certain value, the quenching reactions from N_2_ will surpass the Penning reactions, and then the Penning ionization zone aforementioned will collapse to axis of gas channel making the shape of the plasma transit from the hollow into the contracted solid shape.

**Figure 10:**
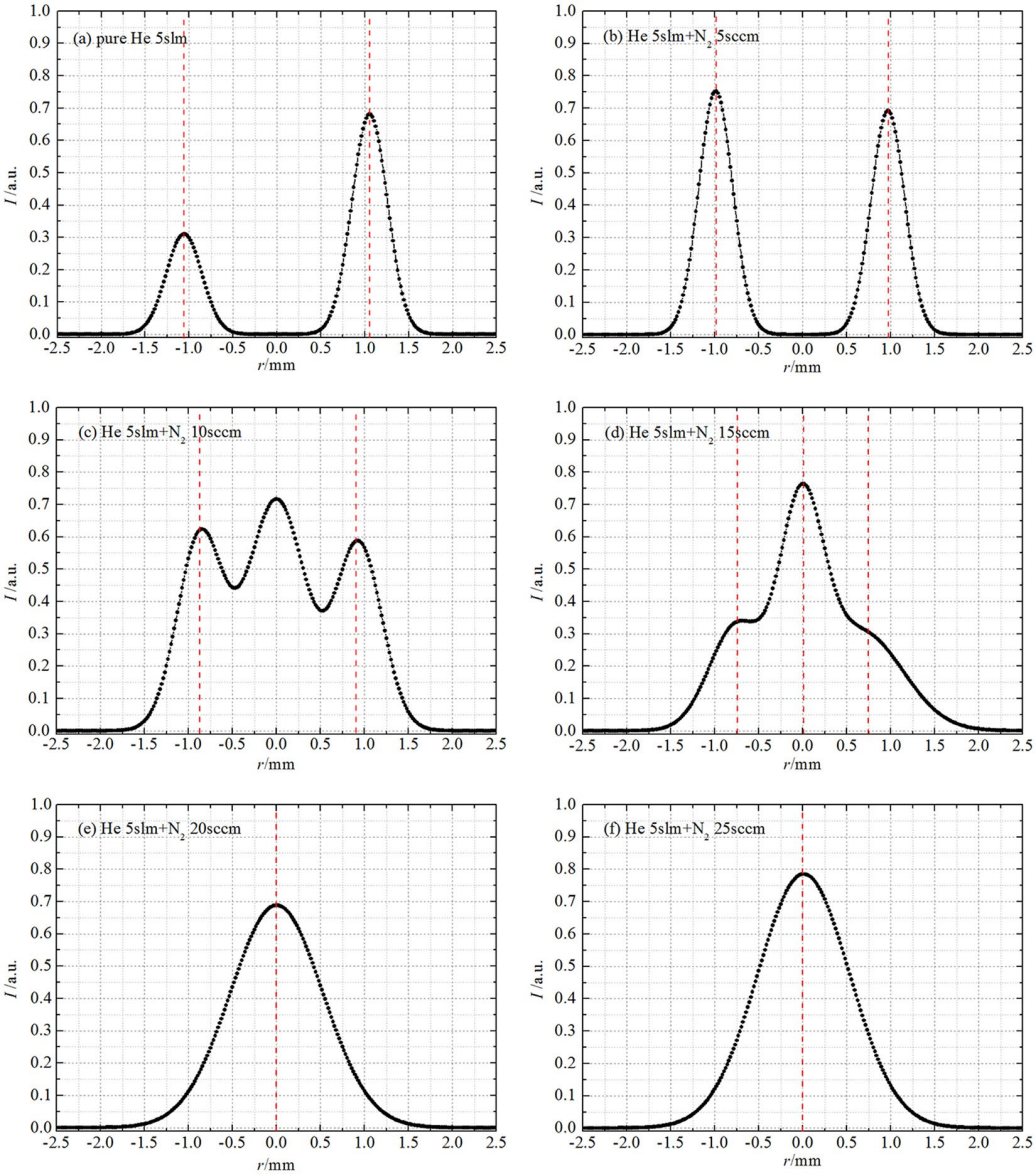
Applied voltage: AC, normalized 1D lightness distribution of front-view ICCD images along radial with different N_2_ ratio: (**a**) N_2_ 0sccm; (**b**) N_2_ 5sccm; (**c**) N_2_ 10sccm; (**d**) N_2_ 15sccm; (**e**) N_2_ 20sccm; (**f**) N_2_ 25sccm.

Further, we extract the lightness data along the direction of 135 degrees on radial photos which lies on right panel in each sub-images. The data extracted were normalized and plotted as 1D curve shown in Fig. 10(a–f). We can clearly see the hollow structure distribution in case of pure He 5slm and He 5slm + N_2_ 5sccm. The peak position of the 1D light curves, representing the most violent excitation and deexcitation reactions, respectively locate at *r* ≈ 1.05 mm, 0.98 mm, 0.93 mm for pure He 5slm (ratio of N_2_ is zero), He 5slm + N_2_ 5sccm (ratio of N_2_ is nearly 0.1 %) and He 5slm + N_2_ 10sccm(ratio of N_2_ is nearly 0.2 %). It means that Penning effect play a dominate role when the ratio of impurity N_2_ is no more than about 0.2 %. At this moment, the ratio of impurity N_2_ in the two zones aforementioned must be higher than mix ratio of N_2_ into pure helium. It agrees well with the numerical simulation results^17^. The quenching effect from N_2_ will over the Penning effect and play a key role when the ratio is larger than about 0.2 %. The quantitative analysis will be further done by mix gas fluid model with or without discharge in future.

So in case of continuous discharge, we can find that the radial optical shape of plasma “bullet” at 10 mm outside nozzle has a dynamic evolution when the ratio of N_2_ and He is changed. It expresses that Penning effect plays a key role for maintaining the hollow structure of plasma “bullet” and its transition. However, its contribution to formation of the hollow structure can not be confirmed only depending on the experiment here.

**Figure 11:**
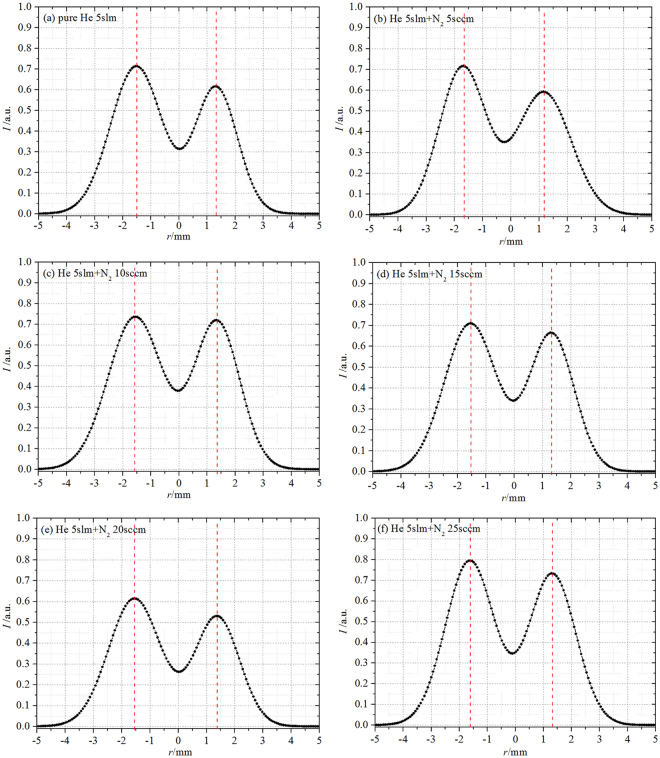
Applied voltage: Pulse, normalized 1D lightness distribution of front-view ICCD images along radial with different N_2_ ratio: (**a**) N_2_ 0sccm; (**b**) N_2_ 5sccm; (**c**) N_2_ 10sccm; (**d**) N_2_ 15sccm; (**e**) N_2_ 20sccm; (**f**) N_2_ 25sccm.

As shown in single pules discharge, the radial image of plasma “bullet” is always hollow structure, not influenced by ratio of impurity N_2_ in present work. The normalized 1D lightness distributions along the direction of 135 degrees on radial ICCD images, in Fig. 11(a–f), also support this point. Two separative peaks have been obtained for each case. The statistic peaks position in all cases is around *r* ≈ 1.4 mm which is larger than that of continuous discharge. In this experiment, metastable He atoms from previous discharge need not to be considered because only single pulse was applied on electrode per discharge. If must be considered, the metastable He atoms should just be generated during the whole pulse. It can be estimated that metastable He atoms, even if produced before discharge and during current pulse, can only move about 3–4 mm during this period driven by gas flow but can not arrive at 10 mm outside the nozzle. That is to say that the “Penning ionization zone” does not present, so that Penning reactions relevant to metastable He atoms can not happen in the interest area. Therefore, it is reasonable to ignore the direct contribution of Penning effect to generating the hollow structure of plasma “bullet”. It means that Penning ionization has no effect on forming the hollow structure of plasma “bullet”.

Naidis^16^, in numerical simulation research, thought that direct collision ionization of electrons at He/Air interface is the main reason for formation of annular “bullet” in development process of discharge plasma. If it’s true, the direct ionization zone should move to axis of plasma jet with increasing N_2_ addition ratio, making radial image of discharge gradually transform into solid. But the results in Figs 8 and 11 are clearly opposite with this explanation.

Except for the factors abovementioned, high breakdown voltage dielectric “tube”, guided laminar gas flow and photo-ionization should be reconsidered for forming the plasma “bullet” with hollow structure. As analyzed, “micro-hybrid interface zone” always exists whatever in high frequency continuous discharge or in single pulse discharge, providing a guide channel for development of discharge. In case of single pulse discharge, discharge ignited from the edge of HV electrode propagates along the surface of inner wall of the tube (see above analysis about laminar gas flow) and goes into the carrier gas flow “tube” continuously. The photo-ionization on head of plasma “bullet” provides advantage for further propagation of discharge^2^ in micro-hybrid interface zone. It should be the most convictive reason for the formation of APPJ hollow structure.

In conclusion, we discussed the formation and evolution of the plasma “bullet” with hollow structure by designing several special experiments. The role of penning effect was also analyzed. Several key conclusions obtained in this paper are listed here: (1) Penning effect plays a minor role in generating hollow structure of plasma “bullet”, but has important contribution to maintaining and evolving the hollow structure when the plasma driven by a continuous discharge. (2) The formation of hollow structure mainly results from guided laminar gas flow and photo-ionization at head of plasma “bullet”. (3) Advanced supposition can be made that the hollow structure of plasma “bullet” should be a characteristic of glow-like APPJ in open ambient. Although we can not provide a strong analysis to support our assumption, we just give one more experiment in Ar/NH_3_ APPJ as an example evidence (Supplementary Information).

## Methods

### Gas mixture and control

In this paper, two kinds of gases with a large flow rate difference, one is high flow rate (such as helium, argon) and another is low flow rate (such as N_2_, NH_3_), need to be mixed before inlet the quartz tube. In order to get homogeneous mixture, we designed single direction hybrid resonator with two channels in and one channel out as well as filled with micro-porous material. Before starting experiment every time, we turn on the gas valves and let gases run over 5 min. After that, power can be turned on to generate discharge and next measurement starts. For low flow rate (like few sccm) and corrosive gas, the special controller with small scale and anti-corrosion material valves is necessary.

### ICCD trigger set and images capture

For AC with 23 kHz discharge, the ICCD images at interest position are easily taken by normal method which has been widely used by researchers. For single discharge, it was a bit complex to capture the ICCDs images at specific position, such as at 10 mm outside nozzle. We employ a relatively unadvanced method here. Two ICCDs are synchronously triggered by voltage and current signals, via setting AB sequence triggering on an LeCroy WaveSurfer 104MXs-B^©^ oscilloscope with 10 GS/s of Sampling rate and 1 GHz of bandwidth. Specifically, condition for “A” event is satisfied → oscilloscope stand-by → condition for “B” event is satisfied → oscilloscope outputs trigger TTL signal → ICCDs response and wait → ICCDs delay time set on software is satisfied → ICCDs act and response signals are monitored by monitor gate and cable. For each case in Fig. 8, many shots (up to 1200) are usual. Two ICCDs should focus on the position 10 mm downstream of nozzle. Among these shots, we should get the right ICCD images at the interest position at least 3 times and select one of them.

## Electronic supplementary material

Supplementary Information
