# Supplementary material for: Plasma “bullet” with hollow structure: formation and evolution
Source: Sci Rep. 2018 May 15;8:7599. doi: 10.1038/s41598-018-25962-z (PMC5953944; doi:10.1038/s41598-018-25962-z)
Supplement: Supplementary file 1 — Supplementary Information [file 41598_2018_25962_MOESM1_ESM.pdf]

Plasma “bullet” with hollow structure: formation and evolution

Zhengshi Chang[*]*, Ni Zhao, Guoqiang Li, and Guanjun Zhang*[*]

Xi’an Jiaotong University, the School of Electrical Engineering, State Key Laboratory of Electrical Insulation and Power Equipment, Xi’an, 710049, PRC

*zschang1984@xjtu.edu.cn; gjzhang@xjtu.edu.cn

**Table of Contents**	**Page #**
Supplementary Content	S2
Supplementary Figure S1	S3
Supplementary Figure S2	S4

SUPPLEMENTARY CONTENT:

An evidence example

For supporting the third conclusion obtained in this paper, a glow-like APPJ in mixture gas of pure argon (Ar) and a little amount of ammonia (NH_3), gotten in our previous work [S1], was employed here. The electrode system and experimental arrangements are the same as Figure 1. Gas flow rate of argon is fixed at 5slm and 3slm respectively in pulse discharge and AC discharge, and that of ammonia has little change. Quartz tube's inner diameter is 5mm in pulse discharge and is 2mm in AC discharge, respectively. The exposure time of the two ICCDs are set as 50ns. The “solid” shape plasma “bullet” can always be obtained when an AC voltage with 23kHz is applied to electrode and the ratio of NH_3 is small, as shown in Figure S1. However, a hollow structure (though not a nice homogeneous ring-shape), was also captured when a single pulse voltage was applied, see Figure S2.

As we know, although more works need to be done about this, it should actually be a good support for our conclusion.

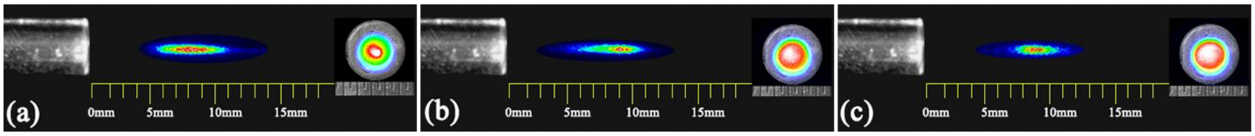

Supplementary Figure S1: APPJ is driven by AC voltage with 23kHz: (a)NH_3 0.4sccm;(b)NH_3 0.6sccm; (c)NH_3 1.0sccm.

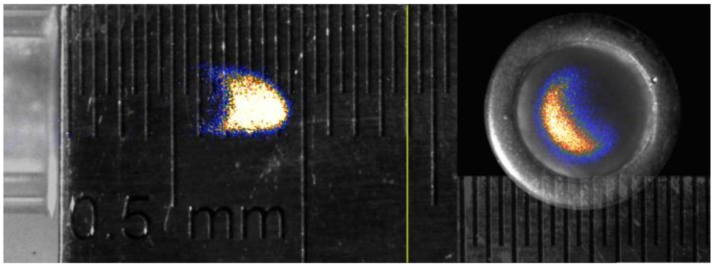

Supplementary Figure S2: APPJ is driven by a pulse voltage with frequency of 0.5Hz and width of 400ms, flow rate of NH_3 is 0.6sccm.
